# CSF1R-related leukoencephalopathy presenting with early apathy, hypoactivity, and cognitive flattening: a case report of a diagnostic challenge

**DOI:** 10.3389/fnhum.2026.1702515

**Published:** 2026-01-21

**Authors:** Wen Yang, Chunli Li, Hongjiang Zhang, Yangjia Zhang

**Affiliations:** 1 Department of Clinical Psychology, The First People's Hospital of Yunnan Province Affiliated Hospital, Kunming University of Science and Technology, Kunming, China; 2 The First People's Hospital of Yunnan Province, Affiliated Hospital, Kunming University of Science and Technology, Kunming, China

**Keywords:** apathy, cognitive impairment, CSF1R, HDLS, hypoactivity

## Abstract

Hereditary diffuse leukoencephalopathy with axonal spheroids (HDLS) is a rare autosomal dominant leukodystrophy primarily caused by mutations in the colony-stimulating factor 1 receptor (CSF1R) gene, characterized by progressive cognitive and motor decline. We present a case of a 42-year-old Chinese woman with a rapidly progressive syndrome featuring prominent apathy, cognitive impairment, and hypoactivity. Brain magnetic resonance imaging (MRI) revealed extensive confluent white matter hyperintensities (Fazekas grade 3) predominantly in frontal and parietal lobes, cerebral atrophy, and thinning of the corpus callosum. Comprehensive genetic testing identified a heterozygous missense mutation in the CSF1R gene (c.2342C > T, p.Ala781Val), located within the tyrosine kinase domain, confirming the Diagnosis of HDLS. This case highlights early apathy and hypoactivity as red-flag manifestations of CSF1R-related leukoencephalopathy in a 42-year-old woman with rapidly progressive cognitive decline. The atypical presentation, initially mimicking psychiatric or demyelinating disease, underscores the need to consider CSF1R sequencing when encountering early-onset cognitive or behavioral deterioration with unexplained white-matter changes, thereby facilitating timely diagnosis and genetic counseling.

## 1 Introduction

Hereditary Diffuse Leukoencephalopathy with Spheroids (HDLS) is a rare neurodegenerative disorder characterized by progressive white matter degeneration, axonal spheroids, and pigmented glia. It is primarily associated with mutations in the CSF1R gene, which encodes the colony-stimulating factor 1 receptor, a critical component in microglial function and brain homeostasis. The disease manifests with a variety of neurological symptoms, including cognitive decline, motor dysfunction, and psychiatric disturbances, often leading to misdiagnosis as other neurodegenerative conditions such as Alzheimer’s disease or frontotemporal dementia (Adams et al., 2018; Wong et al., 2011).

We report a 42-year-old woman carrying a previously reported CSF1R p.Ala781Val variant, who presented with concurrent cognitive impairment, apathy, and hypoactivity. These prominent early behavioral features, together with rapidly progressive cognitive decline and white-matter lesions on MRI, initially led to misdiagnosis as psychiatric or demyelinating disease. This case highlights the diagnostic challenge of CSF1R-related leukoencephalopathy and underscores the importance of considering CSF1R genetic testing in patients presenting with early-onset cognitive and behavioral changes accompanied by unexplained white-matter abnormalities.

## 2 Case presentation

### 2.1 Clinical history

A 42-year-old Chinese woman presented in February 2024 with apathy, hypoactivity, slowed responsiveness, and memory impairment affecting both recent and remote recall. Over the next several months, her symptoms progressively worsened, with the emergence of urinary and fecal incontinence and mood symptoms (mild anxiety and depression). She received symptomatic treatment at local hospitals— including donepezil (5 mg qn), nimodipine (20 mg tid), butylphthalide (0.2 g tid), aspirin (100 mg qd), rosuvastatin (10 mg qn), and later sertraline (50 mg qd)—without clinical improvement. Owing to rapid functional decline, she was referred to our department in June 2025 for further evaluation.

### 2.2 Past and family history

Family aggregation was notable. The patient’s mother and maternal aunt had both developed emotional blunting, hypoactivity, and cognitive decline in midlife, followed by loss of ambulation and dysphagia, and both died within five years of onset. Her younger sister presented with similar symptoms at age 34 and died at 35, while her younger brother (aged 37) recently began showing mild memory decline and disorganized speech.

### 2.3 Neurological examination (June 2025)

Normal cranial nerves, normal tone and strength (5/5), negative Babinski sign, and a mildly broad-based gait. Cognitive testing indicated impairments in recall, orientation, attention, calculation, and visuospatial ability, with MMSE 20/30 and MoCA 13/30.

## 3 Materials and methods

### 3.1 Sample collection and DNA extraction

Genetic testing was performed using a whole blood sample collected from the proband on June 20, 2025. The procedure was approved by the Institutional Review Board, and written informed consent was obtained from the patient and her carers.

### 3.2 Whole exome sequencing (WES)

Whole-exome sequencing (WES) was performed using the Roche KAPA HyperExome kit for DNA capture and enrichment, targeting exonic regions and adjacent splice sites. High-throughput sequencing of the target regions was conducted using the MGISEQ-2000 or DNBSEQ-T7 sequencing platform. The sequencing data underwent quality control to ensure that the average depth of coverage for the target regions was ≥200×, with >98.5% of the target regions having a depth >20 × .

### 3.3 Data analysis

Sequencing reads were aligned to the UCSC hg19 human reference genome using BWA, with duplicates removed. Base quality score recalibration was performed using the GATK tool, and single-nucleotide variants (SNVs), insertions, deletions (Indels), and genotyping were detected. Copy number variations (CNVs) at the exonic level were analyzed using ExomeDepth.

### 3.4 Gene mutation screening and annotation

Gene mutations were screened according to the American College of Medical Genetics (ACMG) guidelines and the American Molecular Pathology (AMP). The pathogenicity of the variants was assessed based on multiple lines of evidence, including literature reports, database comparisons (such as ESP, ExAC, GnomAD), and bioinformatic prediction tools.

### 3.5 Sanger sequencing validation

The CSF1R gene mutation (c.2342C > T, p.Ala781Val) detected in the proband was validated using Sanger sequencing. The mutation was identified as a heterozygous variant, and the validation results were consistent with the findings from whole-exome sequencing.

## 4 Results

### 4.1 Clinical presentation and progression

The 42-year-old female proband initially presented in February 2024 with progressive apathy, decreased verbal output, hypoactivity, and memory impairment affecting both recent and remote recall. Over the following months, her symptoms worsened, accompanied by urinary and fecal incontinence and mood symptoms such as mild anxiety and depression. Neuropsychological assessments documented a progressive cognitive decline: MMSE decreased from 28/30 (2024) to 20/30 (2025), and MoCA from 17/30 (2024) to 13/30 (2025); the 2025 HAMD-17 and HAMA scores were 14 and 9, respectively.

### 4.2 Neuroimaging findings

T2WI/FLAIR revealed multiple punctate and patchy hyperintense signals in the bilateral periventricular regions, corona radiata, centrum semiovale, and corpus callosum. Ventricular system enlargement and widened sulci, cisterns, and fissures were observed. DTI imaging showed sparse but continuous trajectories of the bilateral frontal lobes, periventricular superior longitudinal fasciculus, corticospinal tract, and corpus callosum fibers.

T2WI/FLAIR demonstrated multiple punctate and patchy hyperintense signals in the bilateral periventricular regions, corona radiata, centrum semiovale, and corpus callosum. Compared to the previous scan (November 21, 2024), the lesions have slightly increased in extent. Ventricular system enlargement and widening of sulci, cisterns, and fissures were also noted. DWI revealed a few punctate hyperintense signals in the bilateral periventricular regions and the left centrum semiovale.

### 4.3 Laboratory and CSF analysis

Blood: The total cholesterol is 6.23 mmol/L, low-density lipoprotein (LDL) is 3.71 mmol/L, and fasting glucose is 3.71 mmol/L. The calcium level is 2.09 mmol/L. Tumor markers show carbohydrate antigen 125 (CA125): 52.00 U/L,carbohydrate antigen 724 (CA724): 52.1 U/mL, and prolactin: 2108.2 mIU/L. The triiodothyronine (T3) level is 1.27 nmol/L, the CD4/CD8 ratio is 2.81↑, and the NK cells are 3.73%. Other blood tests, including complete blood count, liver and kidney function, eight items of fluid immunology, C-reactive protein, folic acid, vitamin B12, anti-neutrophil cytoplasmic antibody (ANCA), antinuclear antibody (ANA) profile, antiphospholipid antibody profile, syphilis serology, and HIV tests, all show normal/negative results.

**Figure 1:**
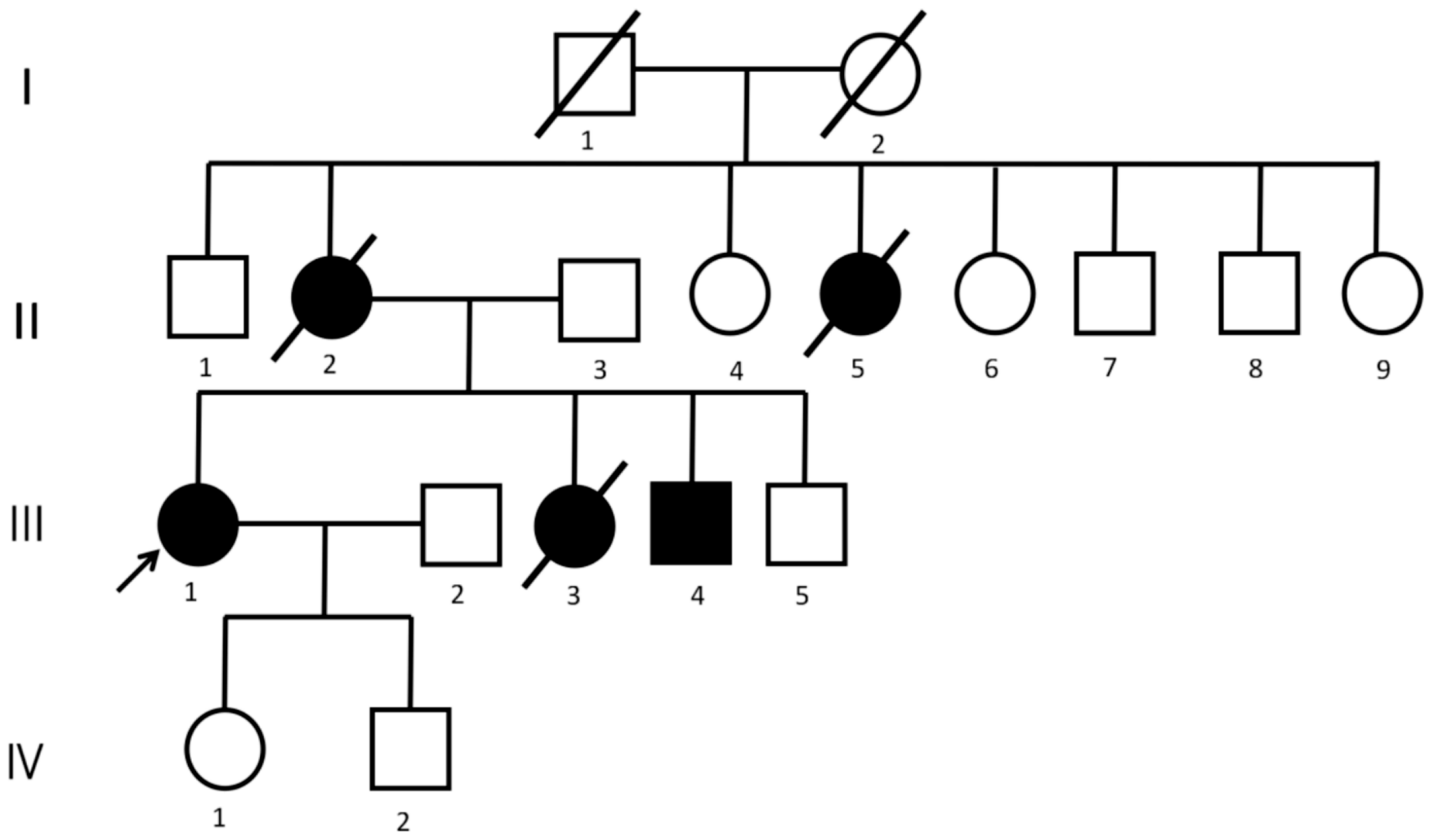
Pedigree of the family. The arrow indicates the proband. The pedigree is constructed based on clinical history reports.

**Figure 2:**
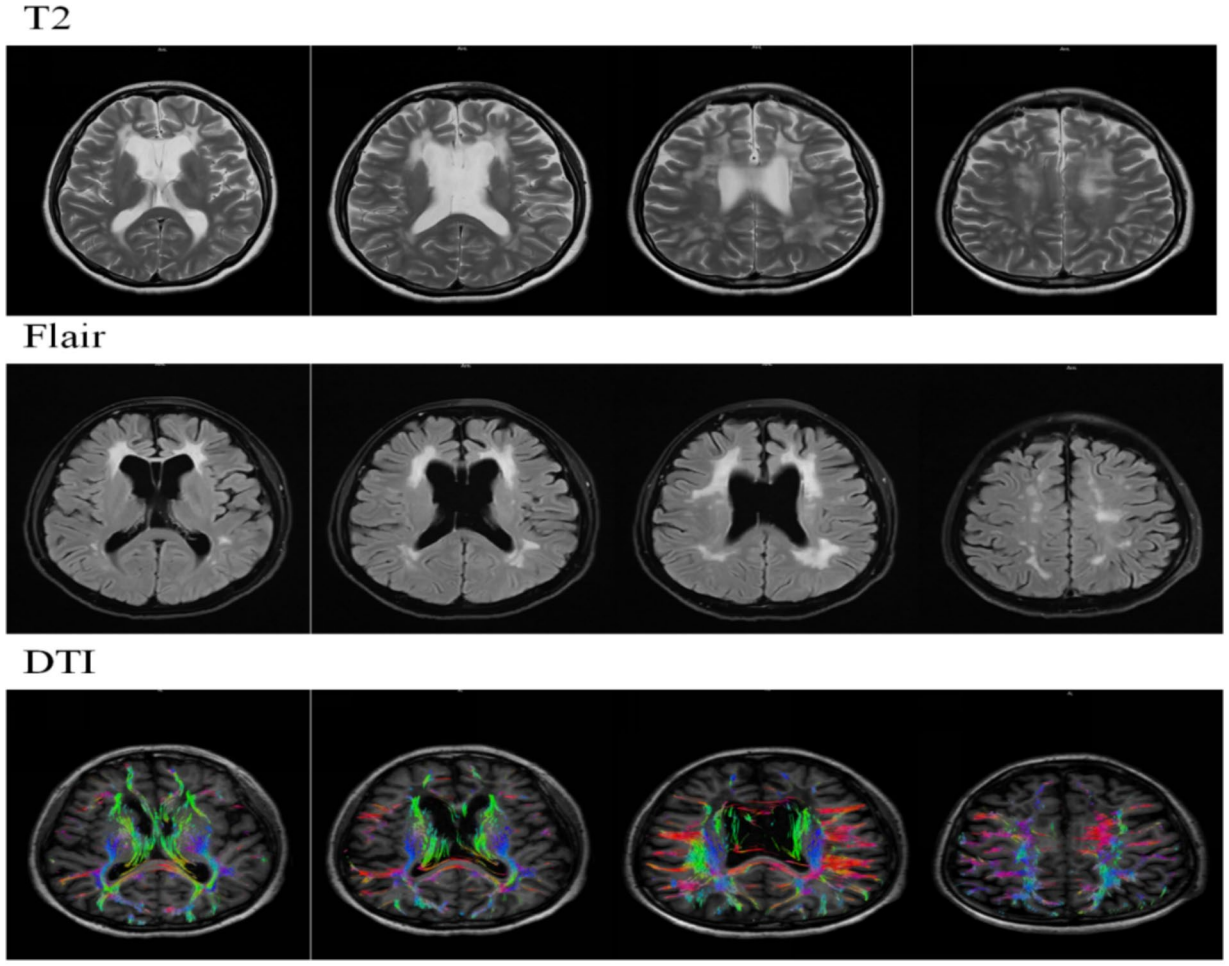
MRI and DTI findings on November 21, 2024.

**Figure 3:**
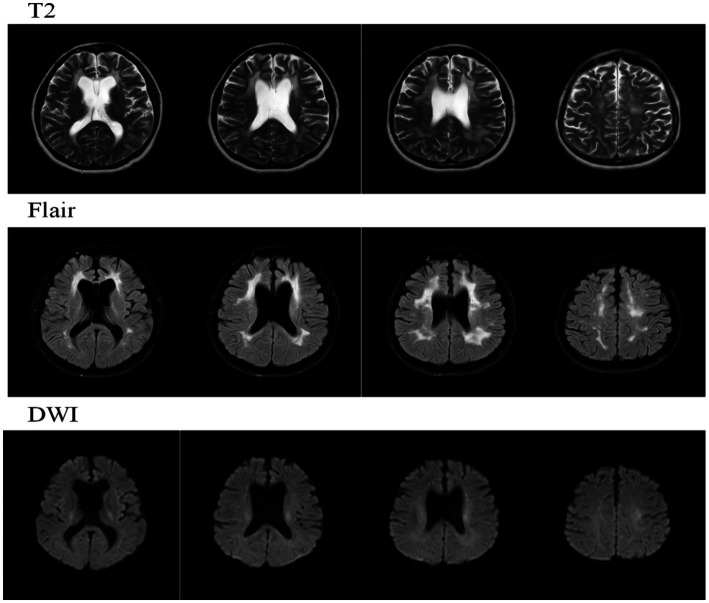
MRI and DWI findings on June 25, 2025.

**Figure 4:**
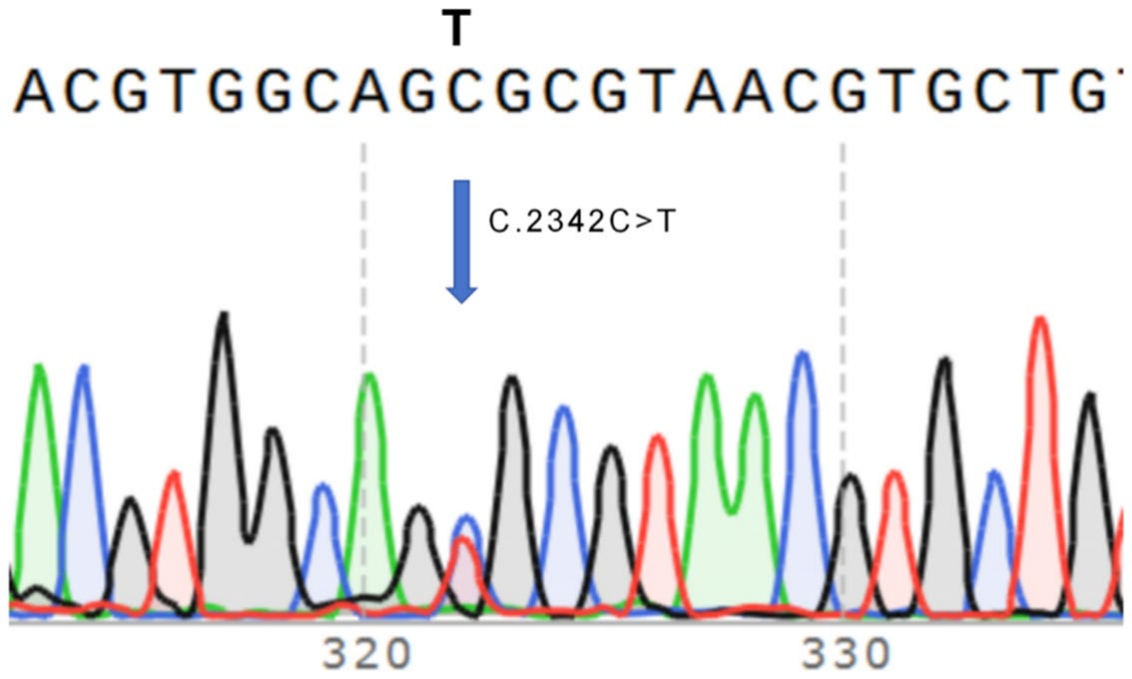
Sanger sequencing electropherogram showing the mutation c.2342C > T.

CSF: Normal pressure/cell count/protein; elevated Aβ1-42 (1,048 pg./mL); The detection of specific antibodies (AQP1, AQP4, MOG, MBP, GFAP, and anti-Flotillin1/2 IgG) revealed no evidence of demyelinating diseases of the central nervous system (Figures 1–4).

### 4.4 Genetic testing and diagnosis

Whole-exome sequencing identified a heterozygous CSF1R c.2342C > T (p.Ala781Val) variant within exon 18 of the tyrosine-kinase domain (NM_001288705.3). Sanger sequencing confirmed this substitution, establishing the molecular diagnosis of CSF1R-related hereditary diffuse leukoencephalopathy with spheroids (HDLS). Considering the patient’s family history—maternal and sibling relatives with progressive cognitive and behavioral decline—the inheritance was deemed autosomal dominant.

This image displays the Sanger sequencing trace of a CSF1R gene mutation, with a straightforward substitution at position c.2342, where cytosine (C) is replaced by thymine (T). The arrow highlights the location of the mutation within the sequence (Tables 1, 2).

**Table 1:** Key MRI and DTI findings over time.

Date	Key MRI findings
Nov 2024	- Confluent WM hyperintensities (Fazekas 3) in frontal/parietal lobes - Corpus callosum thinning - DTI: Reduced integrity of corticospinal tracts
Jun 2025	- Progressive WM lesions extending to the temporal lobes - Septum pellucidum defect with single ventricle formation - No diffusion restriction

**Table 2:** Summary of diagnostic progression, therapeutic interventions, and clinical outcomes.

Date	Diagnostic impression	Therapeutic regimen	Clinical outcome
August 8, 2024 (Local Hospital)	- Early-onset AD (under observation) - Mild cognitive impairment - Suspected demyelinating leukoencephalopathy - Chronic cervicitis - Depressive state - Hyperlipidemia	- Donepezil 5 mg qn - Nimodipine 20 mg tid - Butylphthalide 0.2 g tid - Aspirin 100 mg qd - Rosuvastatin 10 mg qn	No symptomatic improvement after 3 months
November 26, 2024 (Referral Center)	- Demyelinating encephalopathy (Multiple Sclerosis?) - Ischemic cerebrovascular disease - Anxiety-depression syndrome	- Piracetam 0.8 g tid - Donepezil 5 mg qn - Sertraline 50 mg qd - *Ginkgo biloba* extract bid	Progressive deterioration over 7 months
July 17, 2025 (Current Institution)	Definitive Diagnosis: - HDLS (Confirmed CSF1R c.2342C > T) - Key supporting features: • Symmetric frontal–parietal WM lesions (Fazekas 3) • Corpus callosum atrophy • Septum pellucidum defect	Symptomatic support only: - Sertraline 50 mg qd (anxiety) - Physical therapy	Continued decline Prognosis: Severe dementia

## 5 Discussion

In contrast to many cases of CSF1R-related leukoencephalopathy that initially present with cognitive or motor symptoms, the core manifestations in this patient were prominent apathy and psychomotor retardation accompanied by rapidly progressive cognitive decline. These early and predominant neuropsychiatric features, together with characteristic confluent white matter lesions, led to differential considerations of early-onset dementia, multiple sclerosis, or mood disorders prior to genetic confirmation. Notably, despite imaging evidence of white matter involvement, cerebrospinal fluid analysis showed no oligoclonal bands or inflammatory signs, and the periventricular lesions were symmetric and non-enhancing—features inconsistent with a typical primary demyelinating process. This diagnostic trajectory highlights the complexity of CSF1R-related disorders, which can clinically mimic both neurodegenerative and demyelinating diseases. Therefore, in patients presenting with early-onset apathy, progressive leukoencephalopathy unresponsive to conventional therapy, and unremarkable routine investigations, hereditary diffuse leukoencephalopathy with spheroids should be strongly suspected, and prompt CSF1R genetic testing is warranted to establish a definitive diagnosis and to guide appropriate management and genetic counseling (Adams et al., 2018; Wong et al., 2011; Han et al., 2020).

The CSF1R p.Ala781Val variant is located within the tyrosine kinase domain (TKD), a mutational hotspot critical for receptor autophosphorylation and downstream microglial signaling (Pixley and Stanley, 2004). Mutations in this domain, including missense variants, have been implicated in neurodegenerative disorders such as hereditary diffuse leukoencephalopathy with neuroaxonal spheroids (Shi et al., 2019). Recent structural studies demonstrate that CSF1R activation requires symmetric dimerization of its kinase domains, and disease-associated mutations destabilize this dimer, impairing receptor autophosphorylation and signaling fidelity (Zhang et al., 2024). The clustering of pathogenic missense variants, including p.Ala781Val, within the TKD region supports a dominant-negative pathogenetic model, whereby these mutations disrupt kinase dimerization and activity (Schmitz et al., 2024). This dysfunction ultimately compromises microglial homeostasis, as evidenced by studies showing that CSF1R deficiency leads to reduced microglia density and aberrant distribution (Oosterhof et al., 2018), thereby contributing to the loss of white matter integrity. Despite this shared molecular defect, the p.Ala781Val variant exhibits marked phenotypic heterogeneity. Our patient presented with prominent apathy, hypoactivity, and cognitive flattening—features initially suggestive of a psychiatric or early-onset neurodegenerative disorder. In contrast, other reported carriers of the identical variant presented with motor-predominant syndromes: one with motor impairment previously misdiagnosed as primary progressive multiple sclerosis (Codjia et al., 2018), and another with spasticity similarly misdiagnosed as multiple sclerosis (Mochel et al., 2019). This spectrum spanning behavioral, cognitive, and motor domains exemplifies the clinical pleiotropy of a single genetic variant. Nevertheless, neuroimaging provides a unifying diagnostic anchor across this clinical diversity. Brain MRI in all three reported p.Ala781Val carriers demonstrated highly consistent findings: characteristic confluent frontoparietal white matter hyperintensities accompanied by corpus callosum atrophy (Codjia et al., 2018; Mochel et al., 2019). This stable imaging phenotype serves as a pivotal diagnostic clue. Importantly, the disease course driven by this variant demonstrates a uniformly aggressive natural history: one patient deteriorated to a vegetative state within five years (Mochel et al., 2019), the present case showed significant cognitive decline over one year, and another developed severe disability within several years (Codjia et al., 2018). The patient presented with early behavioral and cognitive impairment, atrophy of the corpus callosum, and frontal lobe-predominant white matter lesions, which aligns with the typical clinico-radiological pattern of HDLS: behavioral and cognitive symptoms are often associated with frontal lobe dysfunction, and atrophy of the corpus callosum serves as an important early imaging marker (Stabile et al., 2016; Kondo et al., 2013). The recent identification of a novel pathogenic variant, p.Ala881Val, located in close molecular proximity further underscores the critical role of the TKD; its associated behavioral phenotype (aggression and depression) resonates with the prominent neuropsychiatric presentation observed in our proband, albeit with a different behavioral manifestation (Schmitz et al., 2024). Therefore, integrating the molecular mechanism, diverse clinical manifestations, consistent neuroimaging features, and the rapidly progressive disease course, this case together with the literature highlights that for any adult-onset leukoencephalopathy exhibiting confluent frontoparietal white matter changes and corpus callosum atrophy, CSF1R genetic testing should be pursued as early as possible, irrespective of the clinical phenotype. Early molecular diagnosis is essential to resolve diagnostic uncertainty and to secure a critical therapeutic window for evaluating potential interventions such as hematopoietic stem cell transplantation (Mochel et al., 2019).

This mechanistic understanding bridges the genetic defect to the observed microglial dysfunction. CSF1R encodes a transmembrane tyrosine kinase receptor expressed on microglia that governs their proliferation, survival, and phagocytic activity (Pixley and Stanley, 2004; Sullivan and Pixley, 2014; Ginhoux et al., 2010). Loss-of-function mutations reduce microglial density, leading to impaired clearance of myelin debris and secondary axonal spheroid formation. Recent biochemical evidence further indicates that several HDLS-associated CSF1R variants (e.g., I827A) disrupt symmetric kinase dimerization and can inhibit even the wild-type receptor in trans, providing a mechanistic explanation for the dominant inheritance pattern and profound microglial dysfunction (Zhang et al., 2024). Emerging evidence implicates secondary inflammatory cascades in shaping the clinical phenotype. In a disease model, CSF1R haploinsufficiency upregulated granulocyte colony-stimulating factor (G-CSF), which drove microglial dyshomeostasis and was critically linked to neuropsychiatric and motor deficits (Biundo et al., 2023). Recent single-nucleus transcriptomic analysis demonstrated that pathogenic CSF1R mutations lead to marked microglial depletion and activation-associated lipid accumulation, accompanied by maladaptive astrocyte-oligodendrocyte interactions that impair myelination and white matter maintenance (Du et al., 2025). These insights bridge molecular defects with the observed progressive cognitive and motor decline in patients. Recent experimental models indicate that restoring microglial populations can ameliorate white matter pathology, and integrated transcriptomic evidence supports microglial replacement or CSF1R-targeted gene therapy as promising disease-modifying strategies (Han et al., 2020; Wang et al., 2021; Pan et al., 2024).

Clinically, this case broadens awareness of the CSF1R p.Ala781Val variant in East Asian populations and underscores the imperative of integrating characteristic neuroimaging with genetic testing in the diagnostic workup of atypical leukoencephalopathies. The MRI findings in our patient—including confluent frontal–parietal white matter hyperintensities (Fazekas grade 3), corpus callosum thinning, and a septum pellucidum defect—are consistent with the established imaging phenotype of HDLS (Mickeviciute et al., 2022; Sundal et al., 2012; Bender et al., 2014). Recognition of this disorder at an early cognitive-behavioral stage is crucial, as it facilitates timely genetic counseling for at-risk relatives and allows for enrollment in prospective monitoring or future therapeutic trials. This single-case report cannot establish causality or fully capture the phenotypic variability of CSF1R-related leukoencephalopathy. Longitudinal multicenter registries and functional analyses are needed to clarify mutation-specific mechanisms and to rigorously evaluate potential interventions, including microglial replacement and gene-based therapies (Han et al., 2020; Wang et al., 2021).

## 6 Conclusion

This case report describes a genetically confirmed HDLS proband harboring the pathogenic CSF1R c.2342C > T (p.Ala781Val) mutation, presenting with rapidly progressive apathy, cognitive decline, and hypoactivity. The extensive and confluent white matter lesions with characteristic distribution and atrophy pattern on MRI and negative findings for common mimics were highly suggestive of HDLS; however, a definitive diagnosis relied on genetic testing. This case exemplifies the diagnostic challenges of HDLS due to its clinical overlap with other neurodegenerative disorders and underscores the imperative role of CSF1R gene sequencing in probands with early-onset cognitive/motor decline and unexplained leukoencephalopathy. Given this family’s autosomal dominant inheritance pattern and the confirmed pathogenic variant, genetic counseling is essential for the proband’s offspring and at-risk relatives, each of whom carries a 50% risk of inheriting the mutation. Although no disease-modifying therapy is available, presymptomatic genetic testing, periodic brain MRI (including DTI), and neuropsychological evaluation may allow for early disease progression detection and monitoring. Genetic counseling should also address the psychological and ethical considerations of testing and discuss potential enrollment in future targeted therapies or clinical trials. Early identification of mutation carriers is crucial for personalized surveillance, prognosis assessment, and family planning in HDLS.

## Data Availability

The data supporting the findings of this study are available from the corresponding author upon reasonable request, subject to ethical and privacy restrictions.
